# IMP-38-Producing High-Risk Sequence Type 307 Klebsiella pneumoniae Strains from a Neonatal Unit in China

**DOI:** 10.1128/mSphere.00407-20

**Published:** 2020-07-01

**Authors:** Siyi Wang, Juan Zhao, Ning Liu, Fang Yang, Yiming Zhong, Xiumei Gu, Zijuan Jian, Qun Yan, Qingxia Liu, Hongling Li, Yanming Li, Jing Liu, Hui Li, Liang Chen, Wenen Liu

**Affiliations:** a Department of Clinical Laboratory, Xiangya Hospital of Central South University, Changsha, China; b Molecular Biology Research Center and Hunan Province Key Laboratory of Basic and Applied Hematology, School of Life Sciences, Central South University, Changsha, China; c Center for Discovery and Innovation, Hackensack-Meridian Health, Nutley, New Jersey, USA; d Hackensack Meridian School of Medicine, Seton Hall University, Nutley, New Jersey, USA; Antimicrobial Development Specialists, LLC

**Keywords:** carbapenem-resistant *Klebsiella pneumoniae*, IMP-38, ST307, whole-genome sequencing, plasmid, high-risk clone

## Abstract

An emerging multidrug-resistant Klebsiella pneumoniae high-risk clone of sequence type 307 (ST307) has been increasingly reported worldwide. Here, we described the genomic characteristics of an IMP-38-producing ST307 K. pneumoniae strain and investigated the prevalence of *bla*_IMP-38_ among carbapenem-resistant Klebsiella pneumoniae isolates from a tertiary care hospital in central China. A total of 14 IMP-38-producing ST307 K. pneumoniae strains were identified from 2013 to 2016, with 13 strains isolated from patients with neonatal sepsis in the neonatal ward. PacBio and Illumina whole-genome sequencing analysis performed on a representative IMP-38-producing K. pneumoniae strain, WCGKP294, showed that it contained a circular chromosome and two plasmids. Carbapenemase gene *bla*_IMP-38_ is colocated with *bla*_CTX-M-3_ in transposon Tn*6382* on an IncHI5 plasmid (pWCGKP294-2). WCGKP294 harbors another IncFIB plasmid, pWCGKP294-1, carrying three copies of tandem-repeated IS*26*-*bla*_SHV-2A_-*deoR*-*ygbJ*-*ygbK*-*fucA*-IS*26* composite transposon elements. Phylogenetic analysis placed WCGKP294 in the global ST307 cluster, distant from the U.S. (Texas) and South Africa clusters. Nevertheless, WCGKP294 does not contain the chromosomal fluoroquinolone resistance-associated mutations and IncFIIK/IncFIBK plasmid-associated *bla*_CTX-M-15_ gene that are frequently found in other global ST307 strains.

**IMPORTANCE** We described the genome and resistome characterization of a carbapenem-resistant Klebsiella pneumoniae ST307 strain carrying *bla*_IMP-38_ in China. This report highlights that the high-risk ST307 clone continues to acquire different antimicrobial resistance genes, posing significant challenges to clinical practice, and should be closely monitored.

## Abstract (precis)

We described the genome and resistome characterization of a carbapenem-resistant Klebsiella pneumoniae ST307 strain carrying *bla*_IMP-38_ in China. This report highlights that the high-risk ST307 clone continues to acquire different antimicrobial resistance genes, posing significant challenges to clinical practice, and should be closely monitored.

## INTRODUCTION

Klebsiella pneumoniae is a common cause of both health care- and community-associated infections (1–3). Carbapenems were usually used as last-line antibiotics to treat infections by multidrug-resistant Gram-negative bacteria, including K. pneumoniae. With the widespread usage of carbapenems and the dissemination of carbapenemase-producing Gram-negative pathogens over recent decades, the number of carbapenem-resistant Klebsiella pneumoniae (CRKP) strains increased globally (4), posing a significant challenge to clinical diagnosis, treatment, and infection control.

The global dissemination of CRKP is largely mediated by certain high-risk CRKP strains, such as those of clonal group 258 (CG258), which comprises sequence type 11 (ST11) and ST258 and closely related STs (5). Meanwhile, other high-risk clones contributing to the global spread of CRKP have also been frequently reported. One example is K. pneumoniae ST307, which is an emerging global antimicrobial drug-resistant clone (6–8). In certain regions, ST307 even showed a tendency to replace CG258. A U.S. study sequenced 1,777 extended-spectrum-beta-lactamase (ESBL)-producing K. pneumoniae strains collected over 4 years and found that the CG307 strains caused more infections (36.2%, 644/1,777) than the widely studied CG258 epidemic strains (26.7%, 474/1,777) (9). In addition, a recent study reported a regional spread of OXA-181-producing ST307 CRKP in South Africa (8) that was associated with a promiscuous IncX3 plasmid harboring *bla*_OXA-181_. Moreover, NDM-producing ST307 CRKP strains have also been reported to cause hospital outbreaks elsewhere (10). Those studies indicated that the high-risk ST307 strains, harboring various carbapenemase (*bla*_KPC_, *bla*_NDM_, *bla*_OXA-48_, and *bla*_OXA-181_) and/or ESBL (e.g., *bla*_CTX-M-15_) genes, have spread widely in different regions in the world. However, IMP-producing or VIM-producing ST307 strains remain infrequently reported.

IMP carbapenemase, a member of the metallo-β-lactamases capable of hydrolyzing almost all β-lactams, was first described in Japan in the 1990s (11). In 2011, our group first identified a novel IMP-38 variant in the neonatology department in our hospital (12). In this study, we examined the prevalence of *bla*_IMP-38_-harboirng K. pneumoniae isolates in our hospital and determined their genetic backgrounds. The genomic characteristics of a representative IMP-38-procuding ST307 CRKP clinical strain are further described.

## RESULTS AND DISCUSSION

### Prevalence of IMP-38-producing CRKP and clinical characteristics.

In this study, 14 *bla*_IMP-38_-carrying strains were confirmed by PCR amplification and sequencing from 515 continuously collected K. pneumoniae isolates in a Chinese tertiary hospital from 2013 to 2016. The detection rate of *bla*_IMP-38_ in CRKP isolates from January 2013 to November 2016 was 2.72% (14/515). The yearly frequencies of IMP-38-producing K. pneumoniae clinical isolates were 2.04% (1/49) in 2013, 0.0% (0/82) in 2014, 2.72% (2/88) in 2015, and 3.77% (11/296) in 2016. The results suggested that the IMP-38-producing isolates had persisted in our hospital since the initial identification (12).

Notably, 13 of the 14 IMP-38-positive strains were isolated from patients with neonatal sepsis in the neonatal ward, while the remaining strain was from a patient with persistent pneumonia in the pediatric neurology ward, which is adjacent to the neonatal ward. Specific clinical characteristics are described in Table 1. All 14 patients were preterm infants and had undergone endotracheal intubation, which appeared to be one of major risk factors associated with neonatal sepsis among our patients. We suspected that the infections by IMP-38-producing CRKP might have been associated with endotracheal intubation and might have been caused either by contaminated equipment or by staff contact transmission. Previously published studies also suggested that endotracheal intubation was an independent risk factor for infections caused by multidrug-resistant K. pneumoniae, Acinetobacter baumannii, and Pseudomonas aeruginosa (13–15). However, since these clinical isolates were retrospectively collected, we were unable to recover CRKP from endotracheal tube cultures and therefore cannot confirm whether the presence of IMP-38-producing CRKP was due to endotracheal tube contamination.

**TABLE 1:** Clinical features of patients carrying IMP-38-producing Klebsiella pneumoniae^*a*^

Subject no.	Age (days)	Sex	Specimen source	Sepsis	MIC (mg/liter)											
					TZP	FEP	CRO	CAZ	ATM	IPM	ETP	MEM	AMK	LVX	CIP	SXT
1	0	Male	Blood	Yes	8	≥64	≥64	≥64	≥64	≤1	≥8	≥8	≤2	≤0.25	≤0.25	≤1/19
2	11	Female	Ascites	Yes	≥128	≥64	≥64	≥64	≥64	≤1	≥8	≥8	≤2	≤0.25	≤0.25	≤1/19
3	11	Male	Sputum	Yes	≥128	≥64	≥64	≥64	≥64	≤1	≥8	≥8	≤2	≤0.25	≤0.25	≤1/19
4	30	Female	Blood	Yes	8	≥64	≥64	≥64	≥64	≤1	≥8	≥8	≤2	≤0.25	≤0.25	≤1/19
5	24	Male	Blood	Yes	≥128	≥64	≥64	≥64	≥64	≤1	≥8	≥8	≤2	≤0.25	≤0.25	≤1/19
6	8	Female	Blood	Yes	≥128	≥64	≥64	≥64	≥64	≤1	≥8	≥8	≤2	≤0.25	≤0.25	≤1/19
7	10	Male	Blood	Yes	8	≥64	≥64	≥64	≥64	≤1	≥8	≥8	≤2	≤0.25	≤0.25	≤1/19
8	6	Female	Endotracheal tube	Yes	≥128	≥64	≥64	≥64	≥64	≤1	≥8	≥8	≤2	≤0.25	≤0.25	≤1/19
9	6	Male	Sputum	No	≥128	≥64	≥64	≥64	≥64	≤1	≥8	≥8	≤2	≤0.25	≤0.25	≤1/19
10	28	Male	Stool	Yes	64	≥64	≥64	≥64	≥64	≤1	≥8	≥8	≤2	≤0.25	≤0.25	≤1/19
11	15	Male	Stool	Yes	≥128	≥64	≥64	≥64	≥64	≤1	≥8	≥8	≤2	≤0.25	≤0.25	≤1/19
12	11	Male	Stool	Yes	≥128	≥64	≥64	≥64	≥64	≤1	≥8	≥8	≤2	≤0.25	≤0.25	≤1/19
13	30	Female	Sputum	Yes	≥128	≥64	≥64	≥64	≥64	≤1	≥8	≥8	≤2	≤0.25	≤0.25	≤1/19
14	60	Female	Stool	Yes	≥128	≥64	≥64	≥64	≥64	≥16	≥8	≥8	≤2	≤0.25	≤0.25	≤1/19

a All of the patients were cured of their infections. TZP, piperacillin-tazobactam; FEP, cefepime; CRO, ceftriaxone; CAZ, ceftazidime; ATM, aztreonam; IPM, imipenem; ETP, ertapenem; MEM, meropenem; AMK, amikacin; LVX, levofloxacin; CIP, ciprofloxacin; SXT, trimethoprim-sulfamethoxazole.

### Antibiotic susceptibilities and molecular detection.

All 14 isolates exhibited susceptibility to amikacin, levofloxacin, ciprofloxacin, and trimethoprim-sulfamethoxazole but showed resistance to cefepime, ceftriaxone, ceftazidime, aztreonam, ertapenem, and meropenem, while 13 isolates were susceptible to imipenem (MIC ≤ 1 μg/ml). Ten isolates were resistant to piperacillin-tazobatam (Table 1).

PCR detection of carbapenemase and ESBL genes revealed that all 14 strains carried *bla*_IMP-38_, *bla*_CTX-M-3_, *bla*_SHV-2a_, *bla*_SHV-28_, and *bla*_TEM-1_. Pulsed-field gel electrophoresis (PFGE) analysis showed that these isolates shared highly similar profiles (see Fig. S1 in the supplemental material), suggesting a clonal spread of the same *bla*_IMP-38_-harboring strains.

10.1128/mSphere.00407-20.2 FIG S1 XbaI pulsed-field gel electrophoresis (PFGE) patterns of 11 ST307 K. pneumoniae strains. Three strains were not successfully recovered from the stock and were excluded from the PFGE analysis. The dendrogram was created by the use of unweighted pair group method using average linkages (UPGMA) clustering based on Dice correlation coefficient in BioNumerics software (the positional tolerance settings were as follows: optimization, 1%; tolerance, 1%). Download FIG S1, PDF file, 2.0 MB. Copyright © 2020 Wang et al. 2020 Wang et al. This content is distributed under the terms of the Creative Commons Attribution 4.0 International license.

Multilocus sequence type (MLST) analysis showed that all 14 *bla*_IMP-38_-harboring K. pneumoniae strains belonged to ST307. As mentioned above, ST307 strains have been associated with different carbapenemase genes (*bla*_KPC_, *bla*_NDM_, *bla*_OXA-48_, and *bla*_OXA-181_) and ESBL genes (e.g., *bla*_CTX-M-15_); however, *bla*_IMP_ has not been reported in ST307 strains. To the best of our knowledge, this is the first report of the presence of IMP metallo-β-lactamase in this high-risk clone. To reveal the genomic characteristics of IMP-38-produicng K. pneumoniae ST307 strains, we selected one strain, WCGKP294, for whole-genome sequencing using the combination of PacBio and Illumina HiSeq sequencing.

### Genomic characterization of K. pneumoniae strain WCGKP294.

Strain WCGKP294 was isolated from a sputum sample from a preterm infant in 2016. The combination of PacBio SMRT and Illumina HiSeq sequencing generated the complete closure of the WCGKP294 genome. WCGKP294 harbors a 5,540,574-bp circular chromosome and two plasmids, pWCGKP294-1 and pWCGKP294-2. The WCGKP294 chromosome harbors the *oqxA* and *oqxB* genes, which have been linked to quinolone resistance; the *bla*_SHV-28_ gene, conferring cephalosporin resistance; and the *fosA6* gene, which has been linked to fosfomycin resistance. Strain WCGKP294 carried the same K and O loci (KL102, associated with *wzi* allele 173, and O2v2) as other global ST307 strains, as well as the second putative capsule synthesis locus (Cp2) and a π-fimbria-encoding gene cluster (16). However, inspection of the chromosomal quinolone resistance-determining regions (QRDR) failed to identify amino acid substitutions at GyrA codon 83 or 87 and at ParC codon 80 or 84, which appears to be one of the major genetic differences from other global ST307 genomes (17). Previous study of 95 global ST307 genomes showed that the ParC 80I and GyrA 83I mutations were 100% conserved among all the genomes, with a small cluster of strains carrying an additional GyrA 87N mutation (17). The QRDR mining results in WCGKP294 were in agreement with our susceptibility testing results indicating that all our strains were susceptible to ciprofloxacin and levofloxacin (Table 1).

**FIG 1:**
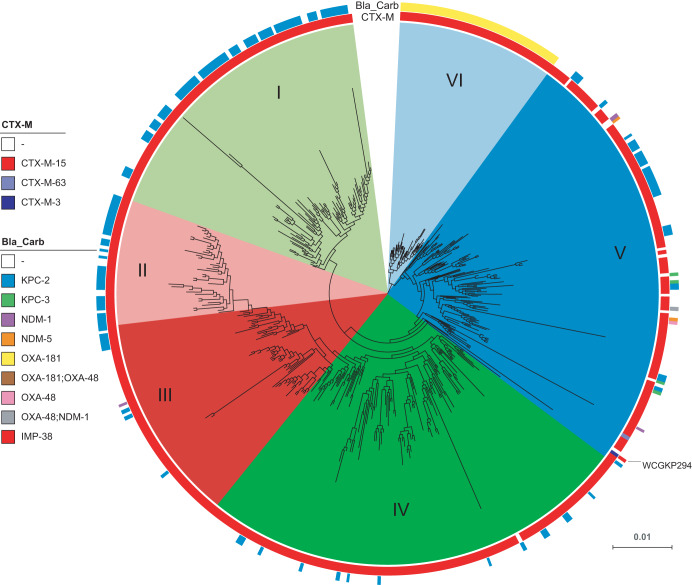
Bayesian phylogenetic analysis of global ST307 K. pneumoniae strains. The analysis included 708 global ST307 genomes from a previous study (6) and the WCGKP294 strain sequenced in the current study. CTX-M, active on cefotaxime; Bla_Carb, carbapenemase genes.

In order to describe the relationship between IMP-38-producing strain WCGKP294 and other global ST307 strains obtained from different countries and sources, a hierarchical Bayesian clustering analysis of ST307 genomes was performed (Fig. 1). Similarly to the results of a previous study (8), the analysis divided ST307 into 6 distinct clades: clades I to VI. Strain WCGKP294 was found to belong to clade V, which is distantly related to clades I to IV (the U.S. Texas clades) and clade VI (the South African OXA-181 clade). Clades I to IV and clade VI represent strains that have been locally isolated over a long period in the United States and South Africa, while the clade V is more like a global lineage and includes isolates from Australia, Brazil, Cambodia, Cameroon, China, Colombia, France, Guinea, Iran, Italy, Nepal, Netherlands, Nigeria, Norway, Pakistan, Thailand, the United Kingdom, and the United States (8).

### Characteristics of *bla*_IMP-38_-harboring plasmid pWCGKP294-2.

**FIG 2:**
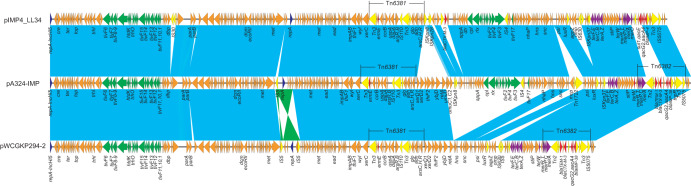
Plasmid structures of pWCGKP294-2 (GenBank accession number CP046614), pA324-IMP (MF344566), and pIMP4-LL34 (CP025964). Light blue shading denotes shared regions of homology of >99% identities, while light green shading indicates reverse-oriented regions of homology. Open reading frames (ORFs) are portrayed by arrows and colored based on predicted gene function. Orange arrows indicate plasmid scaffold regions. The genes associated with the conjugation locus are indicated by green arrows, and replication-associated genes are denoted as dark blue arrows. Antimicrobial resistance genes are indicated by red arrows, while the accessory genes are indicated by yellow arrows.

WCGKP294 was found to contain two plasmids, pWCGKP294-1 and pWCGKP294-2. Plasmid pWCGKP294-1 (GenBank accession number CP046613) was found to be 237,090 bp in length and to belong to the IncFIB incompatibility group, carrying three copies of tandemly repeated IS*26*-*bla*_SHV-2A_-*deoR*-*ygbJ*-*ygbK*-*fucA*-IS*26* composite transposon elements (see Text S1 in the supplemental material). Plasmid pWCGKP294-2 (GenBank accession number CP046614) was found to be 230,910 bp in length with GC content of 47%. Sequence analysis indicated that plasmid pWCGKP294-2 belonged to the IncHI5 incompatibility group (Fig. 2). BLAST analysis showed that pWCGKP294-2 had high similarity to *bla*_IMP-38_-harboring pA324-IMP (MF344566), recovered from a K. pneumoniae strain in Beijing, with 97% query coverage and >99.9% identity, and to *bla*_IMP-4_-harboring pIMP-LL34 (CP025964), isolated from a K. pneumoniae in Chengdu, with 91% query coverage and >99.9% identity (Fig. 2).

10.1128/mSphere.00407-20.1 TEXT S1 Characteristics of plasmid pWCGKP294-1. Download Text S1, DOCX file, 0.01 MB. Copyright © 2020 Wang et al. 2020 Wang et al. This content is distributed under the terms of the Creative Commons Attribution 4.0 International license.

In China, the main IMP type of carbapenemase was found to be IMP-4 (18, 19), which differs from IMP-38 with an amino acid substitution at Ambler position 262 (S262G, serine to glycine) (12). It is likely that IMP-38 evolved from IMP-4 from amino acid substitution. The S262G substitution was previously identified as important for substrate specificity according to studies of other IMP variants, especially IMP-6, which differs from IMP-1 by the same S262G substitution (20–22). The S262G substitution led to a reduction in the imipenem MIC but to higher levels of meropenem and doripenem resistance in IMP-6. We therefore suspected that the imipenem-susceptible but meropenem-resistant profiles found in our IMP-38-producing ST307 CRKP strains may have been due to the S262G substitution (Table 1). Further enzyme kinetic analysis is ongoing to confirm the hypothesis.

Similarly to pA324-IMP, plasmid pWCGKP294-2 had two transposons, designated Tn*6381* and Tn*6382*. The Tn*6381* transposon was located in an ARI-B island, identified as a Tn*6535* derivative, while the Tn*6382* transposon was within an ARI-A island, identified as a Tn*1696* derivative (23). The *bla*_IMP-38_ gene was carried by a class I integron within Tn*6382* with the gene cassette of *bla*_IMP-38_-*qacG2*-*aacA4*-Δ*cat3* on pWCGKP294-2. In addition to *bla*_IMP-38_, several antibiotic resistance genes have also been identified on pWCGKP294-2, including β-lactamase genes *bla*_CTX-M-3_ and *bla*_TEM-1B_, aminoglycoside resistance gene *aacA4*, and chloramphenicol resistance gene *catB3*. In comparison to pIMP4_LL34 and pA324-IMP, pWCGKP294-2 contained an approximately 44-kb deletion that extended from the *umuD* gene to a hypothetical protein gene located upstream of *hns*. This deleted region included the conjugation-encoding *cpl*, *rlx*, and *tivF* genes, which is consistent with the inability of conjugation of pWCGKP294-2 seen during the conjugation experiment (data not shown).

**FIG 3:**
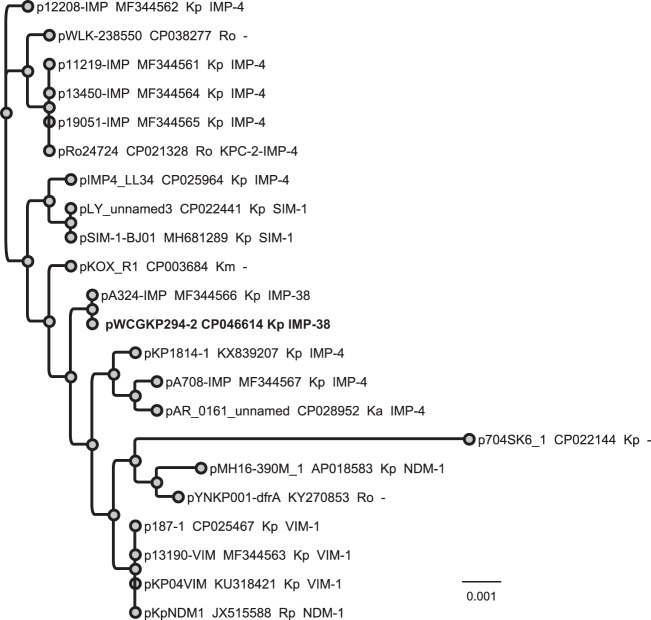
Phylogenetic analysis of 22 IncHI5 plasmids. The tip label was annotated as follows: plasmid name, GenBank accession number, species, and carbapenemase genes. The pWCGKP249-2 plasmid sequenced in this study is highlighted in bold. Kp, K. pneumoniae; Ka, K. aerogenes; Km, *K. michiganensis*; Rp, *R. planticola*; Ro, *R. ornithinolytica*. Hyphens (-) indicate that the plasmids were found to be negative for carbapenemase genes.

A recent study suggested that IncHI5-type plasmid may be another key plasmid vector contributing to the rapid transmission of carbapenemase genes (23). Similarly, our study also suggested that IncHI5 plasmids could serve as a major vector in the spread of *bla*_IMP_ in *Enterobacteriaceae*. To understand the phylogenetic relationship of IncHI5 plasmids, we downloaded 21 additional IncHI5 plasmids from the NCBI database and constructed a phylogenetic tree based on the sequence alignment (Fig. 3). The 22 plasmids were from various strains of K. pneumoniae, K. aerogenes, K. michiganensis, Raoultella ornithinolytica, and R. planticola and had been isolated in different regions in mainland China (*n* = 18), Taiwan (*n* = 1), Viet Nam (*n* = 1), Switzerland (*n* = 1) and the United States (*n* = 1), suggesting the wide distribution of IncHI5 plasmids. Eighteen of the 22 plasmids carried at least one carbapenemase gene, mostly metallo-β-lactamase genes, with the exception of 1 plasmid that coharbored *bla*_KPC-2_ and *bla*_IMP-4_ (pRo24724). The phylogenetic tree showed that pWCGKP294-2 is closest to pA324-IMP, carrying the same *bla*_IMP-38_ in a Tn*6382* element, which is consistent with the BLAST results described above.

Conversely, the previous genomic epidemiological study suggested that the global ST307 strains were closely associated with *bla*_CTX-M-15_. The *bla*_CTX-M-15_ ESBL gene was found in 93.7% of the ST307 genomes (*n* = 89), and among them, 82 (92.1%) carried a pKPN3-307_typeA IncFIIK/IncFIBK plasmid backbone, sharing the same insertion of the IS*Ecp1*-*bla*_CTX-M-15_ transposon within Tn*3* (16). Those results also suggested that *bla*_CTX-M-15_-harboring IncFIIK/IncFIBK plasmids may have coevolved with ST307 strains over time. However, our IMP-38-producing strain, WCGKP294, does not contain *bla*_CTX-M-15_ and instead harbors another *bla*_CTX-M-3_ variant on IncHI5 plasmid pWCGKP294-2 that was found to coexist with *bla*_IMP-38_ within the same Tn*6382* transposon.

In conclusion, we have reported here the identification of IMP-38-producing high-risk ST307 K. pneumoniae strains in a neonatal unit in China. The *bla*_IMP-38_ gene is harbored by a Tn*6382* transposon on an IncHI5 plasmid. Unlike the other global ST307 strains, our IMP-38-producing ST307 strain does not harbor QRDR fluoroquinolone resistance-associated mutations and was not associated with the ESBL *bla*_CTX-M-15_ gene on the IncFIIK/IncFIBK plasmid, suggesting that IMP-38-producing ST307 strains may have a different evolutionary path and are under different antibiotic selection pressures. Nevertheless, our study showed that this high-risk clone continues to acquire various antimicrobial resistance genes, posing significant challenges to clinical practice, and should be closely monitored.

## MATERIALS AND METHODS

### Bacterial isolates and antimicrobial susceptibility testing.

Clinical isolates were collected from January 2013 to November 2016 in a tertiary care hospital in Changsha located in the middle of China. A total 515 unique CRKP isolates (one isolate per patient) were recovered from clinical specimens. MICs of 12 antimicrobial agents were determined for all 515 clinical isolates using the microdilution method, according to the guidelines of the Clinical and Laboratory Standards Institute (CLSI) (24). The study was approved by the institutional review board (IRB) at Xiangya Hospital of Central South University.

### Detection of *bla*_IMP-38_ gene.

All CRKP isolates were screened for the presence of the *bla*_IMP_ gene using a previously described PCR protocol (25–27). PCR amplifications were carried out on an ABI 2720 thermal cycler (Applied Biosystems, USA). The amplicons from IMP-positive strains were then sequenced to determine *bla*_IMP-38_ variants.

### MLST.

MLST was performed on clinical *bla*_IMP-38_-harboring isolates using a method described previously by Diancourt et al. (27). The ST was determined using the database maintained by the MLST Web server (https://pubmlst.org/bigsdb?db=pubmlst_mlst_seqdef).

### Whole-genome sequencing and bioinformatics analysis.

The genomic DNA was extracted from IMP-38-producing ST307 strain WCGKP294 using the SDS method (28). The DNA was subsequently sequenced using single-molecule sequencing (PacBio RS) and an Illumina HiSeq system. The sequencing reads were assembled using Unicycler v0.4.8 (29). The acquired antimicrobial resistance genes were identified using ResFinder 3.0 (30), and the plasmid replicons in the sequenced isolates were identified using PlasmidFinder 2 (31). A core single nucleotide polymorphism (SNP) phylogenetic tree of WCGKP294 and of 708 global ST307 isolates (from 19 countries) was constructed using a method described previously (8). The phylogenetic tree was annotated in iTOL (32). A total of 21 IncHI5 plasmids were downloaded from GenBank, followed by alignment using Mauve 2.4.0 (33). Conserved regions were extracted from Mauve alignment, and a maximum likelihood (ML) phylogenetic tree was generated using FastTree 2.1 (34).

### Data availability.

The complete genome sequences of WCGKP294 were submitted to GenBank under accession numbers CP046612 to CP046614.
