# Supplementary figures and images for: IMP-38-Producing High-Risk Sequence Type 307 Klebsiella pneumoniae Strains from a Neonatal Unit in China
Source: mSphere. 2020 Jul 1;5(4):e00407-20. doi: 10.1128/mSphere.00407-20 (PMC7333572; doi:10.1128/mSphere.00407-20)

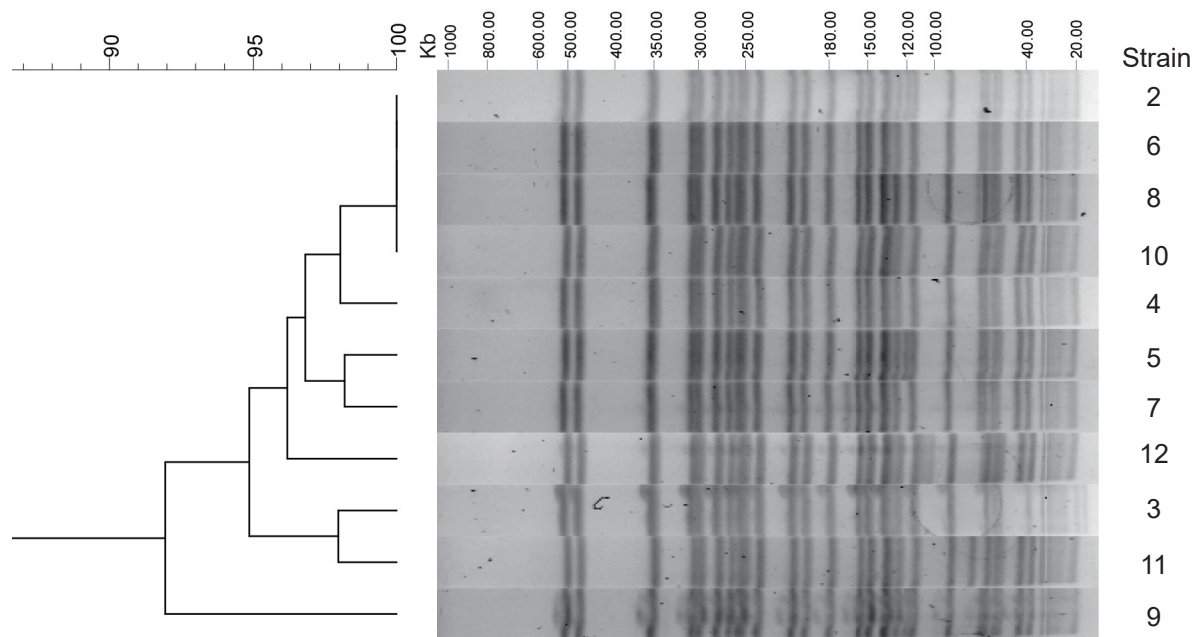

Supplement: FIG S1 [file mSphere.00407-20-sf001.pdf]
