# Supplementary material for: IMP-38-Producing High-Risk Sequence Type 307 Klebsiella pneumoniae Strains from a Neonatal Unit in China
Source: mSphere. 2020 Jul 1;5(4):e00407-20. doi: 10.1128/mSphere.00407-20 (PMC7333572; doi:10.1128/mSphere.00407-20)
Supplement: TEXT S1 [file mSphere.00407-20-s0001.docx]

## IMP-38-producing high-risk ST307 *Klebsiella pneumoniae* strains from a neonatal unit in China

## Siyi Wang^a#^, Juan Zhao^a#^, Ning Liu^a^, Fang Yang^a^, Yiming Zhong^a^, Xiumei Gu^a^, Zijuan Jian^a^, Qun Yan^a^, Qingxia Liu^a^, Hongling Li^a^, Yanming Li^a^, Jing Liu^b^, Hui Li^b^, Liang Chen^c,d^, Wenen Liu^a*^.

**Supplementary data**

**Characteristics of the plasmid pWCGKP294-1**

The plasmid pWCGKP294-1 (CP046613) was 237,090 bp in length with a GC content of 52%. It belongs to IncFIB incompatibility group, with the replicon sequences most close to IncFIB(K)(pCAV1099-114) (CP011596) of 99.64% identities. Interestingly, pWCGKP294-1 contained three copies of extended-spectrum β-lactamases gene *bla*_SHV-2A._ The *bla*_SHV-2A_ genes were located in three tandem repeated IS*26*-*bla*_SHV-2A_-*deoR*-*ygbJ*-*ygbK*-*fucA*-IS*26* composite transposon elements (1), separated by four same oriented IS*26*_._ Similar multiple copies (≥ 3) of IS*26*-*bla*_SHV_-IS*26* like elements have also been found in other plasmids (e.g. CP026154 and LT968767) or chromosome (e.g. CP029738) sequences in *K. pneumoniae*, except for different *bla*_SHV_ variants (*bla*_SHV-12_ or *bla*_SHV-11_). The results suggested that insertion sequences (e.g. IS*26*) mediated recombination contributed significantly to the genomic plasticity in these multi-drug resistant strains.

Blast analysis showed that the sequences of pWCGKP294-1 were close to the sequences of p4 (CM018323, from China) with 97% query coverage and overall 99.9% identities, and plasmid pYML0508_1 (CP045194, from China) with 92% query coverage and overall 99.9% identities, as well as plasmids in strains AR_0161 (CP028952, US), AR_0080 (CP027603, US) and NH25 (CP024875, Thailand) with >88% coverage and overall 99.9% identities. However, pWCGKP294-1 showed very low query coverage (29-39%) to the *bla*_CTX-M-15_-harboirng IncFIIK/IncFIBK pKPN3-307 TypeA (KY271404) and other IncFIBK plasmids (KY271405 - KY271407) previously described in ST307 strains.

**References**

1. **Chen CM, Yu WL, Huang M, Liu JJ, Chen IC, Chen HF, Wu LT.** 2015. Characterization of IS26-composite transposons and multidrug resistance in conjugative plasmids from *Enterobacter cloacae*. Microbiol Immunol 59:516-25.
